# The Value of ^99m^Tc-Methylene Diphosphonate Single-Photon Emission Computed Tomography/Computed Tomography in Detecting Atraumatic Costal Cartilage Fracture in Malignant Tumor Patients

**DOI:** 10.3389/fonc.2020.00138

**Published:** 2020-03-03

**Authors:** Wei Li, Linqi Zhang, Wen Li, Rusen Zhang

**Affiliations:** Department of Nuclear Medicine, Affiliated Cancer Hospital & Institute of Guangzhou Medical University, Guangzhou, China

**Keywords:** costal cartilage, fracture, ^99m^Tc-methylene diphosphonate, single-photon emission computed tomography/computed tomography, malignant tumor

## Abstract

**Objectives:** To assess the clinical significance and single-photon emission computed tomography/computed tomography (SPECT/CT) features of atraumatic costal cartilage fracture (CCF) in patients with malignant tumors.

**Methods:** This was a retrospective review of 38 tumor patients with atraumatic CCF referred to SPECT/CT, who were served as the study group (SG). The features of SPECT/CT of atraumatic CCF were assessed. Another 100 tumor patients who underwent chest SPECT/CT and did not have CCF were randomly selected as the control group (CG). In all patients (SG + CG), the diagnostic powers in the detection of atraumatic CCF were computed among CT, SPECT, and SPECT/CT. The final diagnosis was based on pathological findings and radiologic follow-up of at least 1 year.

**Results:** On SPECT/CT images of atraumatic CCF in the SG, fracture lines, irregular calcification, deformation, and swelling were, respectively, noted in 26.3, 47.4, 34.2, and 18.4% of lesions; low, moderate, and high uptake were, respectively, noted in 13.2, 52.6, and 34.2% of lesions. In all patients (SG + CG), the diagnostic powers in the detection of atraumatic CCF of CT, SPECT, and SPECT/CT were as follows: sensitivity 63.2, 100.0, and 92.1%; specificity 86.0, 81.0, and 94.0%; negative predictive value 86.0, 100.0, and 96.9%; positive predictive value 63.1, 66.7, and 85.4%; and area under the curve value 0.746, 0.905, and 0.931.

**Conclusions:** Atraumatic CCF has certain characteristic appearances on SPECT/CT. It should be enrolled in the differential diagnoses when costal cartilages of patients with malignant tumors show abnormal elevated ^99m^Tc-MDP uptake on scintigraphy. Single-photon emission computed tomography/CT has excellent diagnostic power in detecting atraumatic CCF.

## Introduction

Costal cartilage fracture (CCF) is a rare complication of chest trauma and usually occurs in blunt injuries, which can contribute to rib cage instability. The radiologic findings of bony rib fractures in chest trauma have been widely reported in various literatures (1–3). However, CCF is rarely mentioned. Conventional radiography cannot reveal CCF, unless fractures involve densely calcified cartilages. Some studies (4–7) have proved that computed tomography (CT), magnetic resonance imaging, and ultrasonography are useful in the detection of CCF. However, these imaging evaluations of CCF can often be disturbed by irregular calcification in costal cartilages. The abnormal changes in the distribution of ^99m^Tc-methylene diphosphonate (^99m^Tc-MDP) in calcified costal cartilages caused by fractures can be detected by scintigraphy (8). However, the value of single-photon emission computed tomography/CT (SPECT/CT) in detecting CCF has rarely, if ever, been described in literature.

Patients with malignant tumors often have a high risk of fracture, especially after radiotherapy, chemotherapy, or glucocorticoid therapy (9–14). Kim et al. (13) indicated that patients with breast cancer can lose up to 6.8% of bone mineral density (BMD) during a 3-year follow-up. Stam et al. (14) reported that the risk of rib fracture significantly increased after radiotherapy for lung cancer. Occult fracture can often be accidentally found in patients with cancer without a definite history of trauma. Atraumatic sacral insufficiency fractures can be observed in rectal or cervical cancer after pelvic radiotherapy (10, 11). An atraumatic CCF may be unexpectedly detected by ^99m^Tc-MDP SPECT/CT in patients with malignant tumor, which is occasionally observed in clinical practice. However, there are few previously published reports on CCF or atraumatic CCF in patients with cancer. Currently, the clinical significance and image features of CCF in patients with malignant tumor have not been summarized.

In the present study, we report a series of patients with cancer with atraumatic CCF detected by ^99m^Tc-MDP SPECT/CT, which were routinely performed for the evaluation of bone metastases. This study aimed to assess the clinical significance and SPECT/CT features of atraumatic CCF in patients with malignant tumors.

## Materials and Methods

The Institutional Review Board approval for this retrospective study was obtained from the Ethics Committee.

### Patients

A total of 18,893 patients with tumor underwent ^99m^Tc-MDP whole-body bone scan (WBS) from March 2014 to June 2018 at the Department of Nuclear Medicine. Among them, 2,032 patients had chest SPECT/CT for further evaluation. Of these patients, 38 patients found to have atraumatic CCF were recruited in the present study and served as the study group (SG). Another 100 patients with malignant tumors who underwent chest SPECT/CT and did not have CCF were randomly selected as the control group (CG). The patients with a history of chest trauma were excluded to avoid false-positive findings. In the SG, the clinical data and SPECT/CT features of atraumatic CCF were evaluated. In all patients (SG + CG), the values of detecting atraumatic CCF were compared among CT, SPECT, and SPECT/CT.

### SPECT/CT

All SPECT/CT examinations were performed using a SPECT/CT scanner (Philips Precedence-6, ADAC Laboratories, Milpitas, CA, USA) with dual-head gamma camera system, low-energy high-resolution collimators, and six-slice spiral scanning diagnostic CT. Single-photon emission computed tomography/CT was performed 3 h after intravenous injection of 925 to 1,110 MBq ^99m^Tc-MDP (Atomic High-Tech Co. Ltd., Guangzhou, China). Computed tomography was initially performed, and the parameters were as follows: tube voltage, 140 kV; tube current, 200 mA; window width, 15%; pitch, 1.25; slice thickness, 5 mm; slice space, 3 mm; and matrices, 512 × 512. Single-photon emission computed tomography acquisition protocol was started after CT, and the parameters were as follows: 128 × 128 matrix, 60° angular steps with a range of 180° per gamma camera head, and 30-s projection. Single-photon emission computed tomography/CT fusion images were obtained by JETStream workstation (Philips Medical Systems, Eindhoven, Netherlands). The transverse, sagittal, and coronal planes of CT, SPECT, and SPECT/CT were evaluated, respectively.

### Image Analysis

All CT, SPECT, and SPECT/CT images were independently reviewed and interpreted by three experienced specialists (two nuclear medicine physicians and one diagnostic radiologist). In case of discrepancy, consensus was obtained by a joint reading.

In the SG, the features of SPECT/CT of atraumatic CCF were assessed by the specialists. The following radiologic features of CCF on CT images were evaluated: fracture line, dislocation, irregular calcification, deformation, and swelling. The tracer uptake levels of CCF on SPECT were divided into high, moderate, and low uptake based on whether to be higher than, equal to, or lower than those of the ribs.

In all patients (SG + CG), the examiners were requested to determine the presence or absence of atraumatic CCF. The aforementioned radiologic characteristics of the costal cartilage were considered as positive proofs of CCF on CT. The abnormally increased tracer in the costal cartilage was regarded as positive evidence of CCF on SPECT. The following findings of costal cartilage on SPECT/CT were considered as the criteria for judging the existence of CCF: aforementioned definite CT signs, suspicious CT abnormality with moderate or high uptake, and normal CT finding with high uptake.

The junctions of the costochondral cartilage and sternum-costal cartilage were not included in this analysis, which often represented intense uptake and calcification.

### Diagnosis

A total of 17 patients underwent biopsies and were diagnosed based on pathological results. Of these, 12 patients (SG) were diagnosed with atraumatic CCF, 2 patients (CG) with costal cartilage invasion, and 3 patients with costal chondritis. The other 26 patients in the SG were diagnosed with CCF based on radiologic investigations (SPECT/CT and/or CT) and follow-up of at least 1 year. In the other 95 patients in the CG, the diagnosis of CCF was excluded based on imaging examinations and follow-up of at least 1 year.

### Statistical Analysis

The assessment result of SPECT/CT in the SG was presented as number and frequency (%). The sensitivity, specificity, positive predictive value (PPV), and negative predictive value (NPV) of CT, SPECT, and SPECT/CT in the detection of atraumatic CCF in all patients (SG + CG) were calculated through the McNemar test. Additionally, their diagnostic powers were determined by area under the curve (AUC) value obtained from the receiver operating characteristic (ROC) curve. Continuous data were expressed as means and standard deviations. All data were analyzed by SPSS 13.0 for Windows (SPSS Inc., Chicago, IL, USA).

## Results

### Data on Atraumatic CCF

A total of 38 atraumatic CCF lesions in 38 patients with known cancer were analyzed in the present study. A summary of the clinical characteristics (including age, sex, known cancer, and previous treatment) of all patients is presented in Table 1. There were 15 (39.5%) right- and 23 (60.5%) left-sided CCF lesions. Lesions were most frequently found in costal cartilages 5–7, accounting for 86.8% (33/38) of CCF lesions, 8 in the fifth, 15 in the sixth, and 10 in the seventh costal cartilages. The remaining five lesions were found in the fourth (*n* = 2) and eighth (*n* = 3) costal cartilages. Only 15 CCF lesions (39.5%) showed local symptoms of pain or discomfort. The other 23 lesions (60.5%) remained relatively asymptomatic. In 18 patients, fractures of the bony ribs were noted. There were 13 patients with thoracic bone metastasis and 14 patients with other bone metastases.

**Table 1:** Characteristics of 38 patients with atraumatic CCF.

**Characteristics**	**Value**	**Percentage (range)**
Age (y)	60.5 ± 13.6	43–83
**Sex**		
Male	23	60.53%
Female	15	39.5%
**Known cancer**		
Lung cancer	16	42.1%
Breast cancer	11	28.9%
Esophageal cancer	6	15.8%
Nasopharyngeal carcinoma	1	2.6%
Colorectal cancer	1	2.6%
Liver cancer	1	2.6%
Lymphoma	1	2.6%
Ovarian cancer	1	2.6%
**Previous treatment**		
C	2	5.3%
S + C	1	2.6%
C + R	3	7.9%
S + C + R	3	7.9%
S + C + G	10	26.3%
C + G	5	13.2%
C + R + G	14	36.8%

*S, surgery; C, chemotherapy; R, radiotherapy; G, glucocorticoid*.

### SPECT/CT Features of Atraumatic CCF

**Figure 1:**
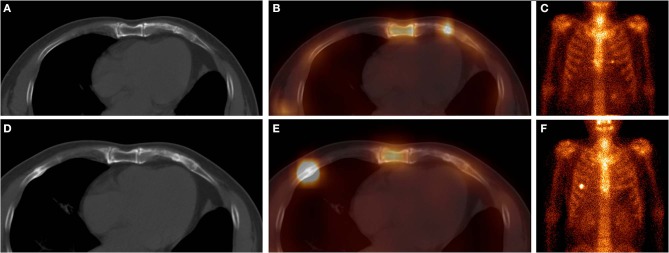
These images were obtained from a 65-year-old man with atraumatic CCF in the SG, who underwent chemotherapy and surgery for left lung adenocarcinoma 3 months ago. ^99m^Tc-MDP SPECT/CT axial images (**A**, CT; **B**, fusion) and WBS image (**C**, anterior) demonstrated a clear fracture line in the calcification of the left fifth costal cartilage, with high-uptake tracer activity. After 9 months, the SPECT/CT images (**D**, CT; **E**, fusion) and WBS image **(F)** showed disappearance of the fracture line of the left fifth costal cartilage, local callus formation, and normal uptake. Moreover, a new focus of intense tracer was noted in the right fifth anterior rib, with callus formation. These indicated that this patient was at a high risk of fracture.

**Figure 2:**
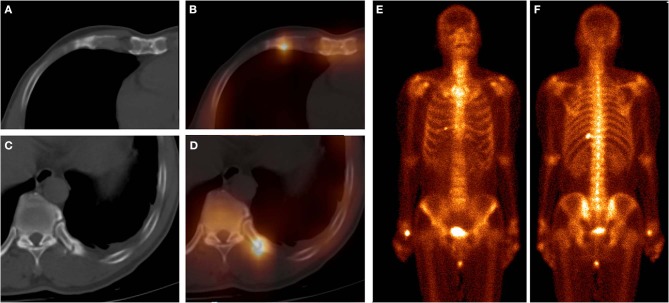
These images were obtained from a 61-year-old man with atraumatic CCF of SG, who underwent chemotherapy for left lung squamous cell carcinoma 5 months ago. ^99m^Tc-MDP SPECT/CT axial images (**A**, CT; **B**, fusion; **C**, CT; **D**, fusion) and WBS image (**E**, anterior; **F**, posterior) demonstrated high-uptake tracer activity in the calcification of the right fifth costal cartilage, with local deformation. Moreover, another focus of intense tracer was noted in the left 11th posterior rib, with fracture line and callus. These indicated that this patient was at a high risk of fracture.

All atraumatic CCF lesions showed abnormal accumulation of ^99m^Tc-MDP on WBS. Single-photon emission computed tomography/CT demonstrated all foci of increased tracer activity in the calcified portions of costal cartilages. A summary of SPECT/CT features in 38 patients with atraumatic CCF is shown in Table 2. The vast majority (24/38 [63.2%]) of CCF lesions demonstrated abnormalities of costal cartilage calcification on CT images. Ten lesions (26.3%) revealed local discontinuities of costal cartilage calcifications with fracture lines (Figure 1). Of these, only three lesions showed mild dislocations and deformations. Irregular calcification was observed in 18 lesions (47.4%). Local deformation of the costal cartilage was noted in 13 lesions (34.2%) (Figure 2). Swelling of the neighboring soft tissues was visible in seven lesions (18.4%). There were 14 lesions (36.8%) without definite abnormality on CT images. Moreover, SPECT images revealed 5 lesions (13.2%) with low uptake, 20 (52.6%) with moderate uptake, and 13 (34.2%) with high uptake.

**Table 2:** Summary of SPECT/CT features in 38 patients with atraumatic CCF.

**SPECT/CT features**	**No. of patients (*n*)**	**Percentage (%)**
**CT**		
Fracture line	10	26.3%
Dislocation	3	7.9%
Irregular calcification	18	47.4%
Deformation	13	34.2%
Swelling	7	18.4%
No abnormality	14	36.8%
**SPECT**		
Low uptake	5	13.2%
Moderate uptake	20	52.6%
High uptake	13	34.2%

### Comparison of Diagnostic Powers

As mentioned previously, of 38 atraumatic CCF lesions in the SG, 24 lesions (63.2%) were diagnosed as positive by CT, and 38 lesions (100.0%) by SPECT. Single-photon emission computed tomography/CT provided an acute diagnosis of CCF in 35 lesions. Three lesions were misdiagnosed as negative by SPECT/CT, which were shown as low uptake with no or mild CT abnormalities.

**Figure 3:**
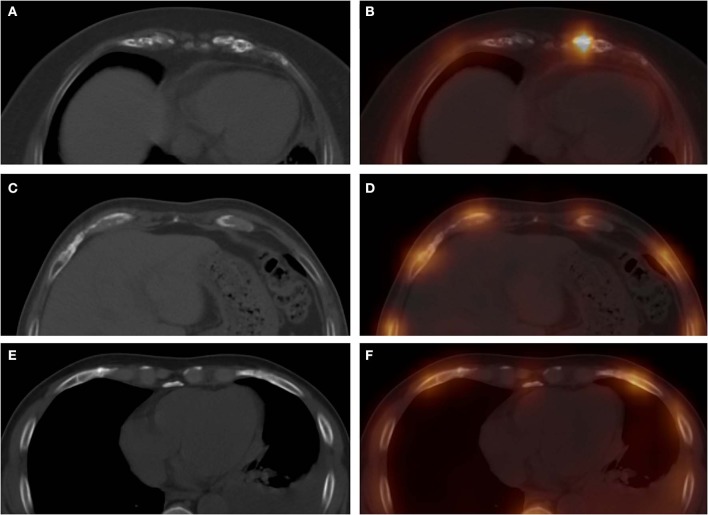
These images were obtained from three patients in the CG. The images (**A**, CT; **B**, fusion) were obtained from a 57-year-old woman with breast cancer, which revealed a high-uptake focus in the left seventh costal cartilage with mild irregular calcification. Computed tomography, SPECT, and SPECT/CT all showed false-positive diagnoses in this patient. The images (**C**, CT; **D**, fusion) were obtained from a 55-year-old man with nasopharyngeal carcinoma, which revealed a moderate-uptake focus in the right eighth costal cartilage with normal calcification. Single-photon emission computed tomography showed a false-positive diagnosis in this patient. The images (**E**, CT; **F**, fusion) were obtained from a 62-year-old man with left lung cancer, which revealed mild irregular calcification in the right sixth costal cartilage, with normal tracer distribution. Computed tomography showed false-positive diagnosis in this patient.

**Figure 4:**
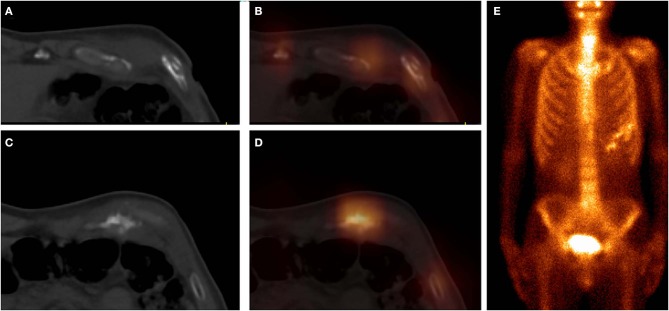
These images were obtained from a 59-year-old patient of CG, who underwent chemotherapy and surgery for esophageal cancer 12 months ago. ^99m^Tc-MDP SPECT/CT axial images (**A**, CT; **B**, fusion; **C**, CT; **D**, fusion) and WBS image (**E**, anterior) demonstrated high-uptake tracer activity in the seventh costal cartilage, with irregular calcification and adjacent soft tissue nodules. The pathological findings confirmed the diagnosis of costal cartilage invasion by chest wall metastasis.

**Figure 5:**
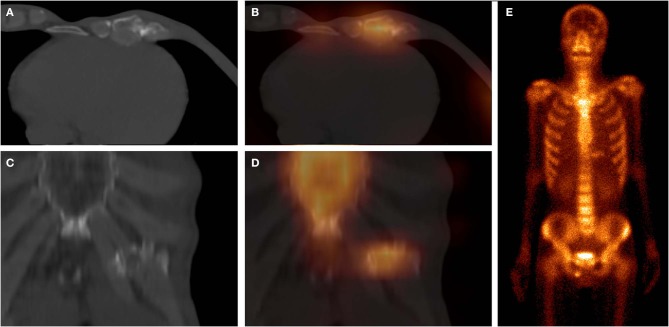
These images were obtained from a 64-year-old patient of CG, who underwent chemotherapy and surgery for esophageal cancer 6 months ago. ^99m^Tc-MDP SPECT/CT axial images (**A**, CT; **B**, fusion), coronal images (**C**, CT; **D**, fusion), and WBS image (**E**, anterior) demonstrated moderate-uptake tracer activity in the sixth and seventh costal cartilages, with irregular calcification and local deformation. The pathological findings confirmed the diagnosis of costal chondritis.

In 100 patients in the CG, 14 patients were misdiagnosed as positive by CT because of radiologic abnormalities, and 19 patients were misdiagnosed as positive by SPECT because of abnormal tracer accumulations (Figure 3). Single-photon emission computed tomography/CT provided incorrect diagnoses in six patients, two patients with costal cartilage invasion (Figure 4), and three patients with chondritis (Figure 5) with obvious CT abnormalities and increased tracer uptake, and one patient with high activity and normal findings on CT.

**Figure 6:**
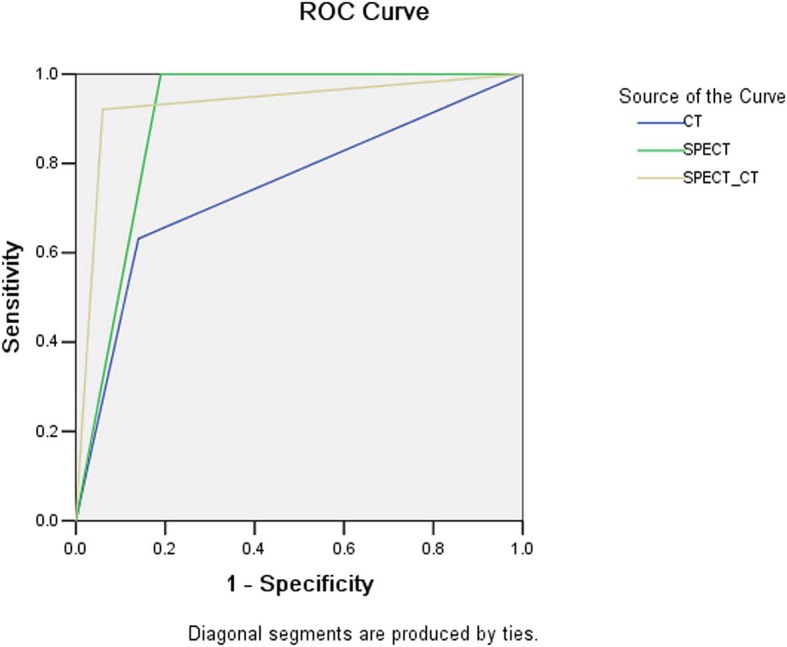
The ROC curves of CT, SPECT, and SPECT/CT in the detection of CCF.

The sensitivity, specificity, PPV, and NPV of CT, SPECT, and SPECT/CT in the detection of atraumatic CCF are presented in Table 3. The results of the ROC curves are shown in Figure 6. The AUC value was the largest in SPECT/CT, followed by SPECT and CT.

**Table 3:** Diagnostic power of CT, SPECT, and SPECT/CT.

**Modalities**	**Sensitivity (%)**		**Specificity (%)**		**NPV (%)**		**PPV (%)**		**AUC value**	
	**Value**	**95% CI**	**Value**	**95% CI**	**Value**	**95% CI**	**Value**	**95% CI**	**Value**	**95% CI**
CT	63.2	46.0–77.7	86.0	77.3–91.9	86.0	77.3–91.9	63.1	46.0–77.7	0.746	0.646–0.846
SPECT	100.0	88.6–100.0	81.0	71.7–87.9	100.0	94.4–100.0	66.7	52.8–78.3	0.905	0.856–0.954
SPECT/CT	92.1	77.5–97.9	94.0	86.9–97.5	96.9	90.6–99.2	85.4	70.1–93.9	0.931	0.874–0.987

## Discussion

Atraumatic CCF is occasionally observed in the clinical practice of patients with high risk of fracture, which is often manifested as incomplete fracture, rarely with fracture line or dislocation of the broken end. It is easy to be missed by clinicians because of the absence of a clear history of trauma. Malignant tumors are always found to be at high risk of fracture, because bone metastases, radiotherapy, chemotherapy, and glucocorticoid therapy can lead to loss of BMD (9–14). However, to the best of our knowledge, no previous study has measured the incidence of atraumatic CCF in malignant tumors. In our series, atraumatic CCF can be observed in 1.9% (38/2,032) of chest SPECT/CT, mainly in patients with lung, breast, and esophageal cancer. The present 38 CCF lesions were all clinically revealed by abnormal ^99m^Tc-MDP uptake. However, there should be some other CCF lesions in 2,032 chest SPECT/CT examinations, which did not reveal increased tracer activity, especially in lesions of old CCF. Unfortunately, those CCF lesions easily had missed diagnosis and were not included in our study or misdiagnosed as degeneration and wrongly attributed to CG because they were asymptomatic and usually have no clear history of trauma and characteristic anatomical changes. Therefore, the patient selection in our study should be a little biased, and the realistic incidence of atraumatic CCF should be >1.9% of our study.

Computed tomography is a common imaging examination for the detection of CCF in clinical practice, which can reveal the fracture line and dislocation (4, 6). Insufficiency fracture of the costal cartilage without a trauma history can be easily misdiagnosed by CT because of mild abnormalities, such as swelling, irregular calcification, and malformation, without typical fracture line or dislocation. In our study, of 38 atraumatic CCF lesions in the SG, only 26.3% (10/38) had fracture lines on CT, 36.8% (14/38) showed slight abnormality, and even 36.8% (14/38) had no abnormal findings. In 100 patients without CCF in the CG of 1, 14 patients (4%) had abnormalities on CT, which included 2 patients with costal cartilage invasion with deformation and swelling, 3 patients with chondritis with irregular calcification or deformation, and the rest with suspicious irregular calcification. Therefore, false-negative and false-positive results can frequently be observed in the CT evaluation of atraumatic CCF.

Costal cartilage fracture is relatively rare in clinical practice, which is mainly found in patients with chest trauma. The location of CCF is mainly determined by the focus of traumatic stress, which can be non-calcified or calcified costal cartilage (15). Atraumatic CCF mostly develops in the high brittle part of the costal cartilage (16). Non-calcified costal cartilage commonly has good elasticity and toughness. However, calcified costal cartilage is often characterized by decreased elasticity and increased fragility. Therefore, the calcified costal cartilage is the most common site of atraumatic CCF. In the present study, all 38 lesions of atraumatic CCF developed in the calcified costal cartilages; although a normal costal cartilage usually does not absorb the tracer of ^99m^Tc-MDP, the calcified part of the costal cartilage can uptake ^99m^Tc-MDP. The fracture occurring in the calcified costal cartilage can cause abnormal accumulation of ^99m^Tc-MDP. Consequently, SPECT has special value in the detection of atraumatic CCF, because it can accurately display the changes in tracer distribution. In this study, SPECT showed 100% sensitivity in atraumatic CCF evaluation, which revealed elevated ^99m^Tc-MDP uptake in all 38 CCF lesions. Most of them were characterized by moderate uptake (52.6%), followed by high uptake (34.2%) and a few as low uptake (13.2%). However, the increased uptake in the costal cartilage can also be observed in metastasis, invasion, chondritis, and calcification physiological uptake (17–19). Hence, SPECT has a certain false-positive result in the assessment of atraumatic CCF. Our results indicated that calcification physiological uptake was the most frequent factor of false-positive result interfering with the diagnostic efficacy of SPECT.

Single-photon emission computed tomography/CT offers the opportunity to obtain both anatomical and functional images, enabling more precise anatomical characterization of SPECT tracer focus using CT component, possibly reducing confusion and enhancing the diagnostic power for atraumatic CCF (20, 21). Calcification physiological uptake is usually characterized by mild increased uptake and normal CT finding, which can be often accurately identified by SPECT/CT. Costal cartilage invasion and chondritis are commonly difficult to be distinguished from CCF on SPECT/CT because of their obvious abnormal CT findings and abnormal tracer activity, while their incidence is extremely low. In our series, SPECT/CT correctly eliminated the majority (68.4% [13/19]) of false-positive findings of SPECT. However, a certain atraumatic CCF can result in a small number of false-negative results on SPECT/CT, which are with mild increased tracer activity combined with normal or suspicious CT findings, especially in minor insufficiency fracture. Therefore, compared with SPECT, SPECT/CT has significantly increased specificity and slightly decreased sensitivity. Through the assessment of AUC values, it is found that the diagnostic power of SPECT/CT is higher than that of SPECT, whereas the power of CT is the lowest.

## Conclusions

Atraumatic CCF has certain characteristics on SPECT/CT. It should be enrolled in the differential diagnoses when costal cartilages of patients with malignant tumors show abnormal elevated ^99m^Tc-MDP uptake on scintigraphy. Single-photon emission computed tomography/CT has excellent diagnostic power in detecting atraumatic CCF.

## Data Availability Statement

This article contains previously unpublished data. All datasets for this study are included in the article.

## Ethics Statement

This study were reviewed and approved by the Institutional Review Board of Affiliated Cancer Hospital & Institute of Guangzhou Medical University. This was a retrospective study, which used clinical data acquired from tumor patients.

## Author Contributions

WeiL and RZ conceived and designed the study. WenL and LZ collected the data. WeiL, RZ, and WenL analyzed the data. WeiL, WenL, and LZ wrote the paper. All authors reviewed the final manuscript.

### Conflict of Interest

The authors declare that the research was conducted in the absence of any commercial or financial relationships that could be construed as a potential conflict of interest.
